# Once-daily delayed-release metformin lowers plasma glucose and enhances fasting and postprandial GLP-1 and PYY: results from two randomised trials

**DOI:** 10.1007/s00125-016-3992-6

**Published:** 2016-05-23

**Authors:** Ralph A. DeFronzo, John B. Buse, Terri Kim, Colleen Burns, Sharon Skare, Alain Baron, Mark Fineman

**Affiliations:** University of Texas Health Science Center, San Antonio, TX USA; University of North Carolina School of Medicine, Chapel Hill, NC USA; Elcelyx Therapeutics Inc., 11975 El Camino Real Suite 305, San Diego, CA 92130 USA

**Keywords:** Clinical care, Clinical study, Lactate, Mechanisms, Metformin

## Abstract

**Aims/hypothesis:**

Delayed-release metformin (Metformin DR) was developed to maximise gut-based mechanisms of metformin action by targeting the drug to the ileum. Metformin DR was evaluated in two studies. Study 1 compared the bioavailability and effects on circulating glucose and gut hormones (glucagon-like peptide-1, peptide YY) of Metformin DR dosed twice-daily to twice-daily immediate-release metformin (Metformin IR). Study 2 compared the bioavailability and glycaemic effects of Metformin DR dosages of 1,000 mg once-daily in the morning, 1,000 mg once-daily in the evening, and 500 mg twice-daily.

**Methods:**

Study 1 was a blinded, randomised, crossover study (three × 5 day treatment periods) of twice-daily 500 mg or 1,000 mg Metformin DR vs twice-daily 1,000 mg Metformin IR in 24 participants with type 2 diabetes conducted at two study sites (Celerion Inc.; Tempe, AZ, and Lincoln, NE, USA). Plasma glucose and gut hormones were assessed over 10.25 h at the start and end of each treatment period; plasma metformin was measured over 11 h at the end of each treatment period. Study 2 was a non-blinded, randomised, crossover study (three × 7 day treatment periods) of 1,000 mg Metformin DR once-daily in the morning, 1,000 mg Metformin DR once-daily in the evening, or 500 mg Metformin DR twice-daily in 26 participants with type 2 diabetes performed at a single study site (Celerion, Tempe, AZ). Plasma glucose was assessed over 24 h at the start and end of each treatment period, and plasma metformin was measured over 30 h at the end of each treatment period. Both studies implemented centrally generated computer-based randomisation using a 1:1:1 allocation ratio.

**Results:**

A total of 24 randomised participants were included in study 1; of these, 19 completed the study and were included in the evaluable population. In the evaluable population, all treatments produced similar significant reductions in fasting glucose (median reduction range, −0.67 to −0.81 mmol/l across treatments) and postprandial glucose (Day 5 to baseline AUC_0–t_ ratio = 0.9 for all three treatments) and increases in gut hormones (Day 5 to baseline AUC_0–t_ ratio range: 1.6–1.9 for GLP-1 and 1.4–1.5 for PYY) despite an almost 60% reduction in systemic metformin exposure for 500 mg Metformin DR compared with Metformin IR. A total of 26 randomised participants were included in study 2: 24 had at least one dose of study medication and at least one post-dose pharmacokinetic/pharmacodynamic assessment and were included in the pharmacokinetic/pharmacodynamic intent-to-treat analysis; and 12 completed all treatment periods and were included in the evaluable population. In the evaluable population, Metformin DR administered once-daily in the morning had 28% (90% CI −16%, −39%) lower bioavailability (least squares mean ratio of metformin AUC_0–24_) compared with either once-daily in the evening or twice-daily, although the glucose-lowering effects were maintained. In both studies, adverse events were primarily gastrointestinal in nature, and indicated similar or improved tolerability for Metformin DR vs Metformin IR; there were no clinically meaningful differences in vital signs, physical examinations or laboratory values.

**Conclusions/interpretation:**

Dissociation of gut hormone release and glucose lowering from plasma metformin exposure provides strong supportive evidence for a distal small intestine-mediated mechanism of action. Directly targeting the ileum with Metformin DR once-daily in the morning may provide maximal metformin efficacy with lower doses and substantially reduce plasma exposure. Metformin DR may minimise the risk of lactic acidosis in those at increased risk from metformin therapy, such as individuals with renal impairment.

***Trial registration*:**

Clinicaltrials.gov NCT01677299, NCT01804842

***Funding*:**

This study was funded by Elcelyx Therapeutics Inc.

**Electronic supplementary material:**

The online version of this article (doi:10.1007/s00125-016-3992-6) contains peer-reviewed but unedited supplementary material, which is available to authorised users.

## Introduction

The glucose-lowering mechanisms of metformin continue to be explored and debated despite 50 years of clinical experience [1]. Until recently, most reports indicated that the antihyperglycaemic actions of metformin were primarily attributable to systemic exposure leading to a reduction in liver gluconeogenesis, with secondary effects including increased insulin-mediated glucose uptake in peripheral tissue [1, 2]. However, there is increasing evidence that at therapeutic doses direct or indirect effects in the gastrointestinal tract explain most, if not all, of metformin’s glucose-lowering actions [3–5].

Reported actions of metformin in the gut include increased secretion of the enteroendocrine L cell hormones glucagon-like peptide-1 (GLP-1) and peptide YY (PYY), possibly through an intestinal 5′AMP-activated protein kinase (AMPK)-dependent pathway, effects on bile acid metabolism and effects on the microbiome [3, 4, 6–8]. A gut-mediated mechanism of action is further supported by the observation that i.v. metformin has an attenuated glucose-lowering effect compared with oral metformin in rats [9, 10], and that i.v. metformin has no acute effects on glucose disposal or hepatic glucose production in humans [11, 12].

Metformin is primarily absorbed from the duodenum and jejunum and is not metabolised; the oral bioavailability of currently available metformin preparations (immediate-release [IR] and extended release [XR]) is ∼50% [13–16]. Thus, approximately half of a typical metformin dose is confined to the gut and delivered to the distal small intestine, where it accumulates in the mucosa at concentrations up to 300 times greater than in plasma [16]. Importantly, even intravenously administered metformin ultimately accumulates in the gut [17, 18], presumably as a consequence of active transport and salivary excretion [15, 17–22]. In mice, i.v. administered [^11^C]metformin was shown to accumulate in the intestinal wall predominantly via organic cation transporters (OCTs) 1 and 2. Intestinal accumulation occurred rapidly (<15 min) and to a much greater extent than in the liver, gall bladder or gastric wall [17]. Because metformin absorption is limited by the transporter rate [13], lower doses have higher bioavailability but are disproportionately less effective than higher doses [23]. This is consistent with the dose–response characteristics of metformin and its weak pharmacokinetic (PK)/pharmacodynamic (PD) relationship [24].

We developed a delayed-release metformin (Metformin DR) formulation designed to release at pH 6.5, which is typically associated with the lower jejunum and ileum, thus bypassing the major sites of metformin absorption. This formulation has been shown to result in ∼50% lower plasma exposure compared with identical daily doses of Metformin IR or Metformin XR [5]. A previous report demonstrated that doses of 600, 800 and 1,000 mg Metformin DR produced significant reductions in fasting plasma glucose (FPG) levels over 12 weeks compared with placebo, with a ∼40% increase in potency compared with Metformin XR [5]. Here, we report the results of two studies into the effects of Metformin DR and Metformin IR on gut hormones and both FPG and postprandial plasma glucose reductions (study 1), and the effects of the Metformin DR treatment regimen (once-daily with the morning or evening meal [once-daily am or once-daily pm] vs twice-daily administration) on metformin PK and PD (study 2).

## Methods

Metformin DR tablets were produced according to current good manufacturing practices and comprised an IR metformin hydrochloride core (Aurobindo Pharma, Hyderabad, India) overlaid with a proprietary enteric coat. The coat delays disintegration and dissolution of the tablet until it reaches a pH of 6.5 in the distal small intestine. Metformin IR treatment used tablets with an identical core but without the enteric coat. Baseline assessments followed identical procedures as those for active treatment but without study medication administration. Both study protocols were conducted in accordance with good clinical practice and approved by the ethics committee of the participating centres (Celerion Inc., Lincoln, NE, and Tempe, AZ, USA). All participants provided written informed consent prior to enrolment. Both studies implemented centrally generated computer-based randomisation using a 1:1:1 allocation ratio.

Eligible participants for both studies were male or female, 19–70 years of age, with a BMI of 25–40 kg/m^2^ and type 2 diabetes mellitus treated with diet and exercise or with metformin and/or a dipeptidyl peptidase-4 inhibitor (DPP-4i). These medications were withheld for at least 14 days prior to randomisation. Baseline characteristics are presented in Table 1.

**Table 1:** Baseline characteristics

Characteristic	Study 1	Study 2
*n*	24	26
Age, years	51.3 ± 10.0	50.9 ± 10.9
Male, %	50	38
Hispanic or Latino, %	58	69
White, black, other^a^, %	79, 8, 13	92, 8, 0
BMI, kg/m^2^	33.3 ± 4.1	31.5 ± 3.2
HbA_1c_, %	7.35 ± 1.11	7.28 ± 1.02
HbA_1c_, mmol/mol	57	56
FPG level, mmol/l	8.94 ± 3.13	9.31 ± 3.17
Prior metformin use, %	71	73
Washout, days	23 (20–26)	23 (16–36)
Prior DPP-4i use, %	0	8
Washout, days	–	22 (16–28)

Data are the mean ± SD or mean (range) for the intent-to-treat population, which includes all randomised participants who received at least one dose of study medication; percentages are based on the number of randomised participants in each study

^a^Other includes Asian, Pacific Islander/Native Hawaiian or American Indian/Alaska Native

### Study 1: PK/PD evaluation of Metformin DR

Study 1 (Clinicaltrials.gov NCT01677299) was a randomised, three-period crossover study with 24 participants. Participants and study site personnel were blinded to treatment assignment. As the tablet number differed between treatment groups (two tablets twice-daily for 1,000 mg doses of Metformin DR and Metformin IR, and one tablet twice-daily for 500 mg Metformin DR), study medication was administered by an unblinded site pharmacist or by study site personnel not involved in study conduct. No other personnel could see the administered drug and study data were blinded until the database was locked. Participants received twice-daily Metformin IR 1,000 mg, Metformin DR 1,000 mg, or Metformin DR 500 mg administered with meals in separate treatment periods. Five-day treatment periods were separated by washout intervals of 9–12 days, depending on participant schedules. At each baseline visit, 16 plasma samples for PD analysis (by Millipore, St. Charles, MO, USA) were collected over 10.25 h starting at t = −15 min from the start of the standardised breakfast at t = 0 h (meal details available in electronic supplementary material [ESM] Table 1). A standardised lunch was served at t = 5 h and study medication dosing began on the evening of the baseline visit. For each period, medication was administered every 12 h until t = −1 min prior to the standardised breakfast on day 5. On day 5, PD samples were obtained at identical time points as those at baseline, and 13 plasma samples for PK analysis (Celerion, Lincoln, NE) were collected over an 11 h sampling period beginning at t = −5 min. Samples for PYY, GLP-1, insulin, glucose and triacylglycerol measurements were collected in K_2_EDTA-containing tubes; and samples for metformin PK were collected in K_3_EDTA-containing tubes.

PK and PD variables were estimated using non-compartmental analysis methods. PK and PD ratios of variables were derived using the evaluable population (i.e. randomised participants who completed periods consistent with protocol procedures). ANOVA was performed on log_*e*_-transformed AUC and maximum plasma concentration (C_max_) for each treatment compared with Metformin IR (PK) or baseline (PD). For PK analysis, models included treatment, sequence and period as fixed effects. For PD analysis, models included treatment day and sequence as fixed effects. For PK and PD, participant nested within sequence was included as a random effect. Log_*e*_-transformed results were back-transformed to the original scale. The 90% CI around the geometric least squares (LS) mean ratios and *p* values are presented.

### Study 2: evaluation of the effect of Metformin DR dosing regimen on metformin PK

Study 2 (Clinicaltrials.gov NCT01804842) was a randomised, three-period crossover study with 26 participants. Participants received one of the following non-blinded treatments during each period: 1,000 mg Metformin DR once-daily am, 1,000 mg Metformin DR once-daily pm or 500 mg Metformin DR twice-daily. The study included three 6–7 day treatment periods (separated by washout periods of 6–12 days, depending on participant schedules). At each baseline visit, participants consumed a standardised lunch (t = −6 h; meal details available in ESM Table 1), dinner (t = 0 h), snack (t = 3 h), breakfast (t = 12 h) and second lunch (t = 18 h), with 34 plasma samples collected over 24 h starting immediately prior to dinner (t = −5 min) for analysis of glucose (Celerion, Tempe, AZ). Once-daily pm and twice-daily medication began after the final (t = 24 h) glucose measurement and immediately prior to the second dinner; once-daily am medication was initiated with breakfast the following morning. At the end of each treatment period, participants performed identical procedures to those performed at baseline, with the addition of 32 plasma samples for PK analysis (Celerion, Lincoln, NE) obtained at first lunch and over the subsequent 30 h period. Urine samples for PK analysis (PharmaNet Canada, Quebec, QC, Canada) were collected during this period at 6 h intervals. Glucose, insulin and PK samples were drawn into sodium fluoride/potassium oxalate tubes, K_2_EDTA-containg tubes, and K_3_EDTA-containing tubes, respectively.

Plasma PK, urine PK and PD variables were determined using non-compartmental analysis methods for the PK or PD intent-to-treat population (randomised participants who received at least one dose). Ratios of PK variables between treatments and ratios of PD variables compared with baseline were derived for participants with complete profiles from at least two treatment periods. ANOVA was performed on the log_*e*_-transformed AUC and C_max_. For PK, models included treatment, sequence and period as fixed effects. For PD, models included pre- vs post-treatment status, sequence and period as fixed effects. For PK and PD, participant nested within sequence was included as a random effect. Log_*e*_-transformed results were back-transformed to original scale. The 90% CI around the geometric LS mean ratios and *p* values are presented.

In both studies, safety and tolerability were evaluated through assessment of adverse events, concomitant medication use, clinical laboratory values and vital signs. All treatment-emergent adverse events (TEAEs) were attributed to the most recent treatment prior to onset.

## Results

### Study 1: PK/PD evaluation of Metformin DR

All 24 participants received at least one treatment dose of the study regimen. Demographic and baseline characteristics (Table 1) were similarly distributed across treatment sequences. Three participants had adverse events leading to their withdrawal (one had a serious gastrointestinal stromal tumour, two had vomiting) and two participants discontinued early for personal reasons. All 19 participants who completed the study were considered evaluable (ESM Fig. 1).

**Fig. 1:**
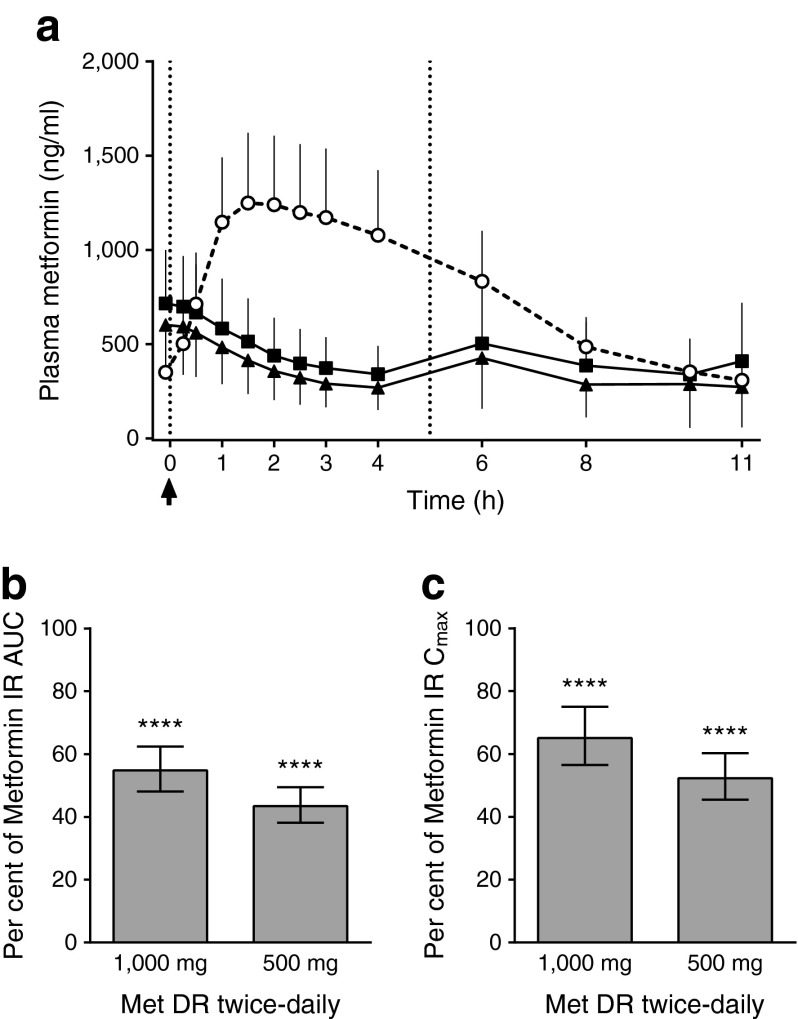
Study 1: plasma metformin concentrations and bioavailability. (**a**) Steady state plasma metformin concentrations (mean ± SD) by treatment. White circles, 1,000 mg Metformin IR twice-daily; black squares, 1,000 mg Metformin DR twice-daily; black triangles, 500 mg Metformin DR twice-daily. All treatments were administered at t = −1 min (arrow) relative to the standardised breakfast (t = 0 h) and lunch (t = 5 h) (dotted vertical lines). (**b**, **c**) Plasma metformin relative bioavailability and exposure at steady state (data are the geometric LS mean and 90% CI for the ratios of twice-daily 1,000 mg Metformin DR [Met DR] and 500 mg Metformin DR to twice-daily 1,000 mg Metformin IR). Evaluable population, *n* = 19. ****p < 0.0001 vs twice-daily 1,000 mg Metformin IR

Figure 1a presents the mean ± SD PK concentrations by treatment at day 5. After the morning Metformin IR administration, metformin concentrations rapidly increased to peak at 1.5 h; in contrast, following administration of both doses of Metformin DR, concentrations continued to decrease to 4 h; at 6 h there was a small rise to a level <40% of the peak level observed with Metformin IR. Plasma metformin concentrations for the 1,000 mg Metformin DR dose were higher than for the 500 mg dose at all time points, but the dose–response relationship was not linear. Figure 1b,c presents the relative exposure for twice-daily 500 mg and 1,000 mg Metformin DR vs twice-daily 1,000 mg Metformin IR at steady state (day 5). Compared with 1,000 mg Metformin IR, 1,000 mg and 500 mg Metformin DR resulted in significantly lower exposures (AUC, −45% and −57%, respectively). Peak concentrations were also significantly lower for 1,000 mg (−35%) or 500 mg (−48%) Metformin DR compared with 1,000 mg Metformin IR.

**Fig. 2:**
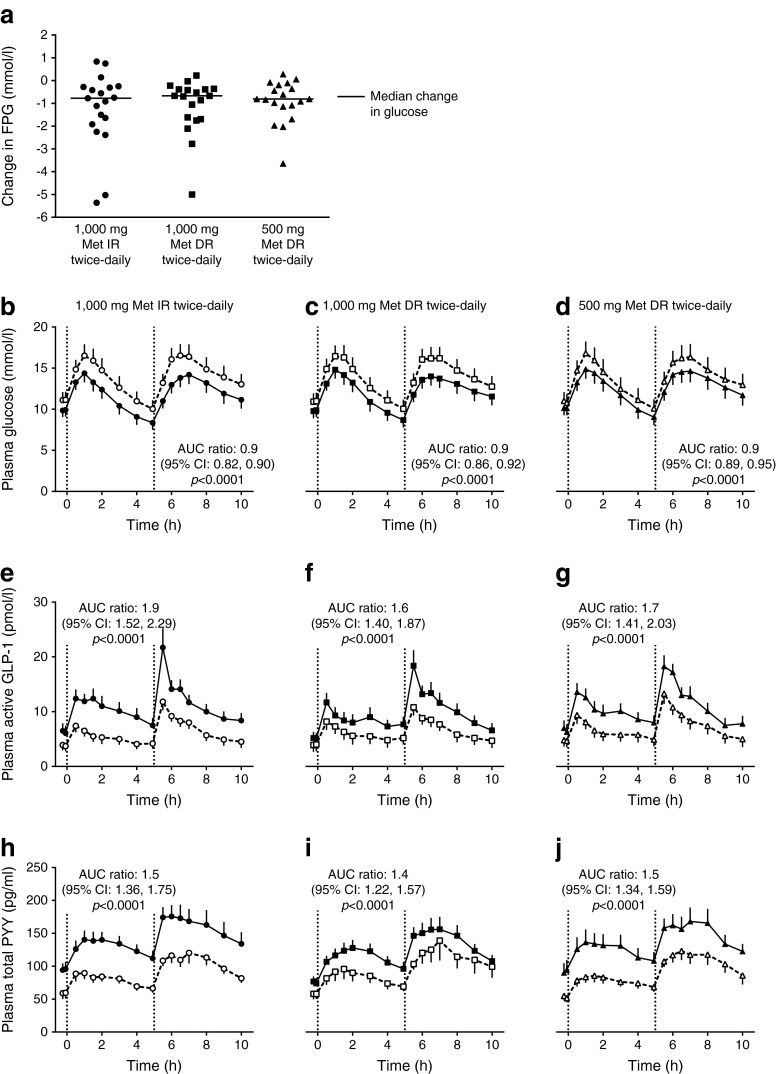
Study 1: change in plasma glucose and gut hormone levels. (**a**) Individual (median) changes from baseline to day 5 in FPG concentrations by treatment. (**b**–**d**) Mean and SEM for plasma glucose at baseline and day 5. (**e**–**g**) Mean and SEM for plasma GLP-1 active at baseline and day 5. (**h**–**j**) Mean and SEM for PYY total at baseline and day 5. White symbols, values at baseline; black symbols, values at day 5. Evaluable population, *n* = 19. All treatments were administered at t = −1 min relative to the standardised breakfast (t = 0 h) and lunch (t = 5 h; dotted vertical lines). Met, metformin

Figure 2a presents the median and individual participant changes in FPG from baseline to day 5 by treatment. LS mean reductions at day 5 were similar among treatment groups and significantly different from baseline (*p* < 0.01 for all) as follows: −1.25 mmol/l for 1,000 mg Metformin IR, −1.11 mmol/l for 1,000 mg Metformin DR and −0.91 mmol/l for 500 mg Metformin DR (all twice daily; Table 2). All treatments significantly decreased plasma glucose AUC at day 5 by 10% (Fig. 2b–d). There were no significant changes from baseline in insulin AUC or peak concentrations (data not shown). Figure 2e–j present the mean plasma GLP-1 and PYY concentration profiles at baseline and day 5 by treatment and time point. Baseline plasma GLP-1 and PYY concentrations were similar among treatments. All metformin treatments significantly increased gut hormone AUC at day 5 by 62–87% for GLP-1 and by 38–55% for PYY. Although not pre-specified or powered for between-group comparisons, there was no significant difference in nominal *p* values between each Metformin DR treatment arm and Metformin IR in the gut hormone response. Fasting plasma GLP-1 and PYY concentrations also were significantly increased at day 5 for each treatment (Table 2).

**Table 2:** Study 1: change in pharmacodynamic variables

Variable	1,000 mg Met IR twice-daily (*n* = 19)	1,000 mg Met DR twice-daily (*n* = 19)	500 mg Met DR twice-daily (*n* = 19)
FPG, mmol/l			
Baseline	11.13 ± 0.90	10.94 ± 0.93	11.04 ± 0.97
End of treatment	9.88 ± 0.90	9.84 ± 0.93	10.12 ± 0.97
LS mean difference from baseline	−1.25 ± 0.38	−1.11 ± 0.28	−0.91 ± 0.21
*p* value	0.0040	0.0009	0.0004
Fasting plasma GLP-1 Active (pmol/l)			
Baseline	3.79 ± 1.16	3.93 ± 1.19	4.73 ± 1.31
End of treatment	6.32 ± 1.16	5.10 ± 1.19	6.62 ± 1.31
LS mean difference from baseline	2.53 ± 0.83	1.17 ± 0.54	1.89 ± 0.45
*p* value	0.0067	0.0444	0.0005
Fasting total plasma PYY (pg/ml)			
Baseline	59.30 ± 9.67	57.50 ± 7.92	53.14 ± 10.81
End of treatment	95.05 ± 9.67	75.36 ± 7.92	91.80 ± 10.81
LS mean difference from baseline	35.75 ± 6.30	17.87 ± 6.07	38.66 ± 9.89
*p* value	<0.0001	0.0087	0.0010

Data are the LS mean ± SEM for the evaluable population

### Study 2: effect of Metformin DR dosing regimen on metformin PK/PD

All 26 participants received at least one dose of study medication. Two participants withdrew from the study due to adverse events (vomiting). Two participants were not included in the PK and PD intent-to-treat populations because they left the study before the end of the first treatment period. Twelve randomised participants completed all treatment periods included in the protocol procedures and were included in the evaluable population (ESM Fig. 2). Of those participants excluded from the evaluable population, six did not complete the study and eight were non-compliant with the dosing schedule.

**Fig. 3:**
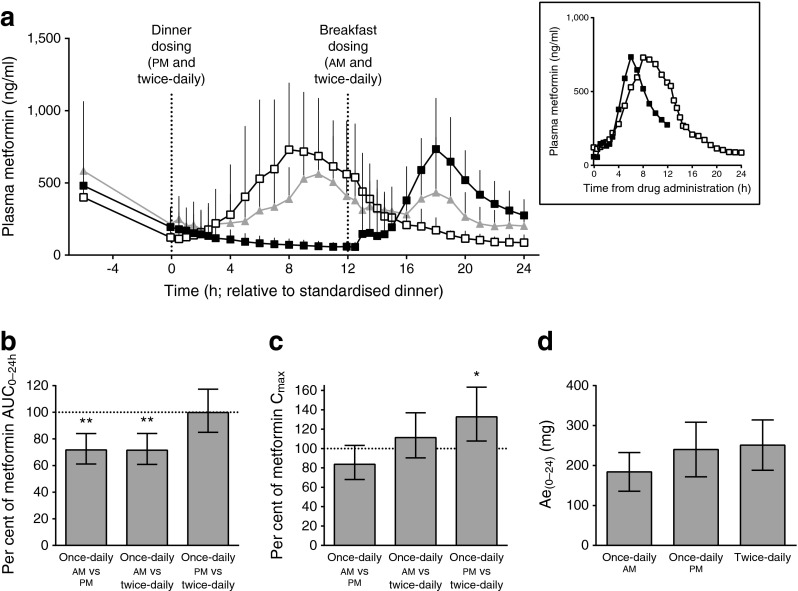
Study 2: change in metformin concentrations and bioavailability. (**a**) Steady state plasma metformin concentrations (mean ± SD) by treatment. Inset: mean plasma metformin concentration from the time of dose administration (t = 0). Black squares, 1,000 mg Metformin DR once-daily am; white squares, 1,000 mg Metformin DR once-daily pm; grey triangles, 500 mg Metformin DR twice-daily. (**b**, **c**) Plasma metformin (mean and 90% CI) relative bioavailability and exposure at steady state. (**d**) 24 h urine excretion (Ae) of metformin (mean and %CV) by treatment at steady state. Evaluable population, *n* = 12. Meals were administered at t = 0, 3, 12 and 18 h. **p < 0.01, *p < 0.05

Figure 3a presents the mean ± SD plasma metformin concentrations at steady state by treatment and time point. Peak plasma concentrations following once-daily pm dosing were right-shifted compared with those for once-daily am dosing, suggesting that the start of metformin absorption was delayed following an evening dose compared with a morning dose. Total metformin exposure over 24 h at steady state was significantly lower (by approximately 30%) for once-daily am dosing relative to once-daily pm and twice-daily dosing (Fig. 3b). The metformin peak concentration was 33% higher for once-daily pm dosing relative to twice-daily dosing (Fig. 3c). The mean metformin excretion in urine over 24 h at steady state was lower for once-daily am than for once-daily pm and twice-daily Metformin DR, consistent with the reduced bioavailability of Metformin DR once-daily am (Fig. 3d).

**Fig. 4:**
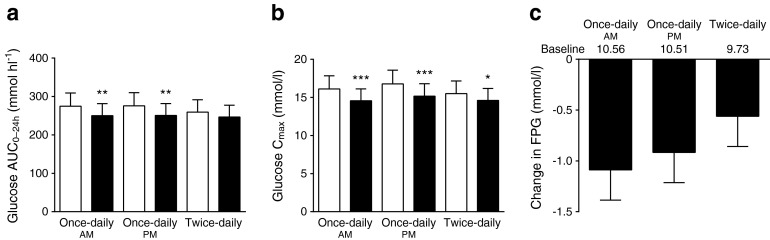
Study 2: change in plasma glucose. (**a**, **b**) Overall plasma glucose and peak exposure at baseline and steady state. White bars, baseline; black bars, treatment. (**c**) FPG change from baseline at steady state. Data are the geometric LS mean and SEM (**a**, **b**) or LS mean and SEM (**c**) for the evaluable population (*n* = 12). *p < 0.05, **p < 0.01, ***p < 0.001

The baseline glucose concentrations were reasonably well matched between both once-daily dose groups, but the baseline value for the twice-daily group was modestly lower (Fig. 4a). Both once-daily dosing regimens resulted in similar, significant decreases in plasma glucose AUC_0–24h_ of 9% after meal challenges from baseline, despite a 30% decrease in total metformin plasma exposure with once-daily am vs once-daily pm dosing (Fig. 3b). Twice-daily dosing showed a trend (*p* = 0.099) for decreased plasma glucose from a baseline of 5%. The mean FPG was decreased at the end of treatment for all three treatment groups; although no significant differences were observed among treatments (Fig. 4c), reductions were greatest following the regimen with the lowest plasma metformin concentrations (once-daily am).

### Safety and tolerability

Table 3 displays the TEAEs with a >5% incidence in any treatment arm. Consistent with metformin prescribing information, TEAEs were primarily gastrointestinal. In study 1, diarrhoea was reported across all treatment groups, while nausea (*n* = 2), vomiting (*n* = 2) and retching (*n* = 1) occurred only with Metformin IR. Two participants withdrew because of the TEAE of vomiting with Metformin IR. Of note, all gastrointestinal events with onset after 500 mg Metformin DR dosing (diarrhoea, *n* = 2; dyspepsia, *n* = 1; abdominal pain, *n* = 1) occurred at least 10 days after the last dose of the study treatment, suggesting that they were unrelated to treatment. Aside from diarrhoea (reported in Table 3), no additional gastrointestinal adverse events occurred with 1,000 mg Metformin DR.

**Table 3:** TEAEs with a >5% incidence in any treatment arm

TEAE	Study 1: double blind			Study 2: non-blinded		
	1,000 mg Met IR twice-daily (*n* = 22)	1,000 mg Met DR twice-daily (*n* = 20)	500 mg Met DR twice-daily (*n* = 20)	1,000 mg Met DR once-daily am (*n* = 23)	1,000 mg Met DR once-daily pm (*n* = 24)	500 mg Met DR twice-daily (*n* = 23)
Any TEAE	6 (27)	5 (25)	4 (20)	6 (26)	5 (21)	7 (30)
Gastrointestinal disorder	5 (23)	3 (15)	2 (10)	3 (13)	2 (8)	4 (17)
Diarrhoea	3 (14)	3 (15)	2 (10)^a^	2 (9)	0 (0)	1 (4)
Nausea	2 (9)	0 (0)	0 (0)	0 (0)	0 (0)	2 (9)^b^
Vomiting	2 (9)	0 (0)	0 (0)	0 (0)	0 (0)	2 (9)^b^
Nervous system disorder	5 (23)	1 (5)	1 (5)	0 (0)	0 (0)	4 (17)
Dizziness	3 (14)	0 (0)	0 (0)	0 (0)	0 (0)	1 (4)
Headache	2 (9)	1 (5)	1 (5)	0 (0)	0 (0)	1 (4)
General disorder and administration site conditions	0 (0)	0 (0)	1 (5)	1 (4)	3 (13)	3 (13)
Vessel puncture site pain	0 (0)	0 (0)	0 (0)	1 (4)	0 (0)	2 (9)^a^

Data are *n* (%)

^a^Both events occurred >10 days after the last dose of study medication

^b^One of the two events occurred >10 days after last dose of study medication

Met, metformin

In study 2, the most common adverse events with non-blinded Metformin DR were gastrointestinal events. Two participants had the TEAE of vomiting, leading to withdrawal from the study after twice-daily treatment (one of these events occurred 11 days after the last dose of study medication). There were no clinically meaningful differences in vital signs, physical examinations or laboratory values.

## Discussion

Metformin is the most commonly prescribed oral glucose-lowering medication, and is recommended as first-line therapy by most diabetes professional societies if well tolerated and not contraindicated because of renal impairment [25]. Despite decades of research and clinical experience, the primary site and mechanism of action of metformin is still under debate. The fact that metformin is not metabolised in vivo and is 50% bioavailable [13] leads to comparable gut and plasma exposures with typical dosing, along with substantial metformin accumulation in the intestine [16]. Nonetheless, the vast majority of studies have focused on its direct effects on the liver [26–29], although typically at suprapharmacological concentrations. In contrast, recent investigations have explored the gut-mediated actions of metformin, including effects on gut hormones that regulate glycaemia and satiety, bile acid secretion, and the gut microbiome [3, 4, 6–8, 30]. For example, Duca et al showed that duodenal, but not portal, metformin infusion lowered blood glucose in insulin-resistant rats through a GLP-1 dependent mechanism [4]. Specifically, they proposed that metformin enhances GLP-1 secretion from intestinal L cells that bind and activate GLP-1 receptors on the afferent vagus nerve, leading to reduced hepatic glucose production through a gut–brain–liver axis. In addition, we recently reported results from a 12 week clinical study showing an apparent 40% increase in potency when metformin is delivered to the ileum via Metformin DR compared with Metformin XR: half of the latter is absorbed into the circulation, with the remainder travelling to the distal small intestine [5].

This report provides data that increases our understanding of the pharmacology and gut effects of metformin. Our results demonstrate that the metformin effect of reducing both fasting and prandial glucose is largely the result of its action on a target(s) in the lumen of the distal small intestine, rather than the result of high systemic exposure. Although once-daily and twice-daily Metformin DR dosing reduced blood glucose to similar extents, once-daily am dosing resulted in the lowest daily metformin exposure. This is consistent with accumulation in the small intestine, rather than plasma exposure, being the primary determinant of the glycaemic response. These findings could also explain why achieving a maximal glucose-lowering effect with current metformin requires repeated dosing and why discontinuation of metformin treatment leads to a gradual, rather than an immediate, loss of glucose control [31]. This study did not assess effects on other putative mechanisms of gut-mediated improvement in glycaemic control with metformin (e.g. bile acid effect, microbiome effects); therefore, the relative contributions of gut hormones on glycaemic improvement for the two treatments cannot be fully discerned. Nevertheless, the nearly identical improvements in glycaemic control observed with Metformin IR and Metformin DR suggest that gut-mediated mechanisms are responsible for the overwhelming majority of glycaemic improvement observed at clinically relevant doses.

In study 1, the rate and extent of metformin exposure were significantly reduced for 1,000 mg Metformin DR twice-daily compared with 1,000 mg Metformin IR twice-daily. Despite the differences in metformin exposure, all treatments resulted in similar, significant reductions in fasting and postprandial glucose, as well as increases in GLP-1 and PYY from baseline to day 5. Twice-daily 500 mg Metformin DR was almost as effective as Metformin IR in reducing plasma glucose and enhancing gut satiety hormones, suggesting that a total daily dose of 1,000 mg metformin delivered directly to the ileum may be close to the maximally effective dose. The observed increases in GLP-1 are well within the range required to produce salutary effects on glucose homeostasis [32]. The reduction in body weight or mitigated weight gain observed with metformin also may result from alterations in gut hormones such as PYY [33]. Of note, these hormones may have additive or synergistic effects on metabolic endpoints such as glucose control and appetite [34, 35].

In study 2, Metformin DR once-daily dosing regimens resulted in similar, significant decreases in plasma glucose after meals compared with baseline (Fig. 4). The mean FPG was also significantly decreased with all three regimens compared with baseline, with the numerically greatest reduction observed with once-daily am treatment. The slightly smaller glucose reduction observed with twice-daily treatment may reflect lower average fasting and postprandial baseline glucose values for that treatment group (Fig. 4a,b,c). Total metformin exposure was lower with once-daily am dosing than with once-daily pm or twice-daily dosing. Slowed gastrointestinal transit during sleep [36] following pm dosing may allow prolonged metformin contact with gut transporters which modestly increases absorption relative to morning dosing. Indeed, the right-shifted PK profile with Metformin DR pm dosing compared with am dosing (together with previous findings that Metformin DR pm dosing results in peak concentrations approximately 10 h post-dose [5]) is probably responsible for the elevated am concentrations observed with Metformin DR vs Metformin IR observed in study 1.

The adverse event profile observed in study 1 suggests similar or improved tolerability for Metformin DR vs Metformin IR, particularly with regard to upper gastrointestinal tolerability. No nausea or vomiting was reported with either Metformin DR dose; in contrast, two participants discontinued because of vomiting with Metformin IR. Diarrhoea appeared to be dose dependent, with a 14–15% incidence for twice-daily 1,000 mg Metformin IR and Metformin DR, but no events within 10 days of the final dose for twice-daily 500 mg Metformin DR. Importantly, given the apparent increase in potency of Metformin DR compared with current metformin formulations, lower total daily doses are the future clinical target [5]. Metformin DR was also well tolerated in study 2 participants: only one participant reported nausea/vomiting within 10 days of treatment. However, as the study was not blinded, no definitive conclusions can be made based on that data.

Metformin DR may ultimately provide a unique treatment option for patients with renal impairment owing to its targeted delivery to the distal small intestine and consequent lower systemic exposure compared with current metformin. Specifically, metformin-associated lactic acidosis (MALA) is a particular concern in patients with impaired renal function [37]. MALA is a rare [37–39] but life-threatening condition usually associated with high (>5 μg/ml) systemic metformin concentrations secondary to overdose or reduced renal clearance. Even in patients with mild or moderate renal impairment, a concurrent event that further reduces metformin clearance and/or alters lactate metabolism (e.g. sepsis, reduced tissue perfusion, anoxia, impaired hepatic metabolism) may increase the MALA risk [40]. In these individuals, reducing metformin exposure by lowering the dose (as is common practice with drugs that act peripherally and are renally cleared) leads to both a disproportional increase in bioavailability and a reduction in ileum exposure [13], which results in a marked reduction in efficacy [23, 41].

In summary, compared with Metformin IR, delivery of metformin to the ileum with Metformin DR at the same or lower doses resulted in comparable glucose lowering and hormone secretion, but significantly lower systemic exposure and a similar number or fewer gastrointestinal events. Once-daily Metformin DR administered in the morning resulted in the lowest systemic bioavailability while maintaining the glucose-lowering effect obtained with twice-daily dosing. These data provide substantial evidence that currently prescribed metformin predominantly works in the gut. Based on its gut-restricted properties, Metformin DR could provide a means of administering metformin to patients for whom it is currently contraindicated (e.g. those with renal impairment) and to others who cannot tolerate currently available metformin formulations.

## Electronic supplementary material

Below is the link to the electronic supplementary material.

ESM (PDF 260 kb)

## Abbreviations

AMPK: 5′ AMP-activated protein kinase
C_max_: Maximum observed plasma concentration
DR: Delayed release
DPP-4i: Dipeptidyl peptidase-4 inhibitor
FPG: Fasting plasma glucose
GLP-1: Glucagon-like peptide-1
IR: Immediate release
LS: Least squares
MALA: Metformin-associated lactic acidosis
OCT: Organic cation transporters
PD: Pharmacodynamic
PK: Pharmacokinetic
PYY: Peptide YY
TEAE: Treatment-emergent adverse event
XR: Extended release
