# Supplementary material for: Once-daily delayed-release metformin lowers plasma glucose and enhances fasting and postprandial GLP-1 and PYY: results from two randomised trials
Source: Diabetologia. 2016 May 23;59:1645–54. doi: 10.1007/s00125-016-3992-6 (PMC4930485; doi:10.1007/s00125-016-3992-6)
Supplement: Supplementary file 1 — (PDF 260 kb) [file 125_2016_3992_MOESM1_ESM.pdf]

ESM Table 1. Standardized Meal Details (Study 1 and Study 2)

	Kilojoules (Kcals)	Carbohydrate	Fat	Protein
Study 1				
Breakfast (t=0 h)	2,197 (525)	58%	22%	20%
Lunch (t=5 h)	4,434 (1,060)	58%	32%	10%
Study 2				
Lunch (t=-6 h)	4,434 (1,060)	58%	32%	10%
Dinner (t=0 h)	3,132 (749)	40%	44%	16%
Snack (t=3 h)	787 (188)	91%	9%	0%
Breakfast (t=12 h)	2,151 (514)	59%	30%	11%
Lunch (t=18 h)	4,434 (1,060)	58%	32%	10%

ESM Fig. 1

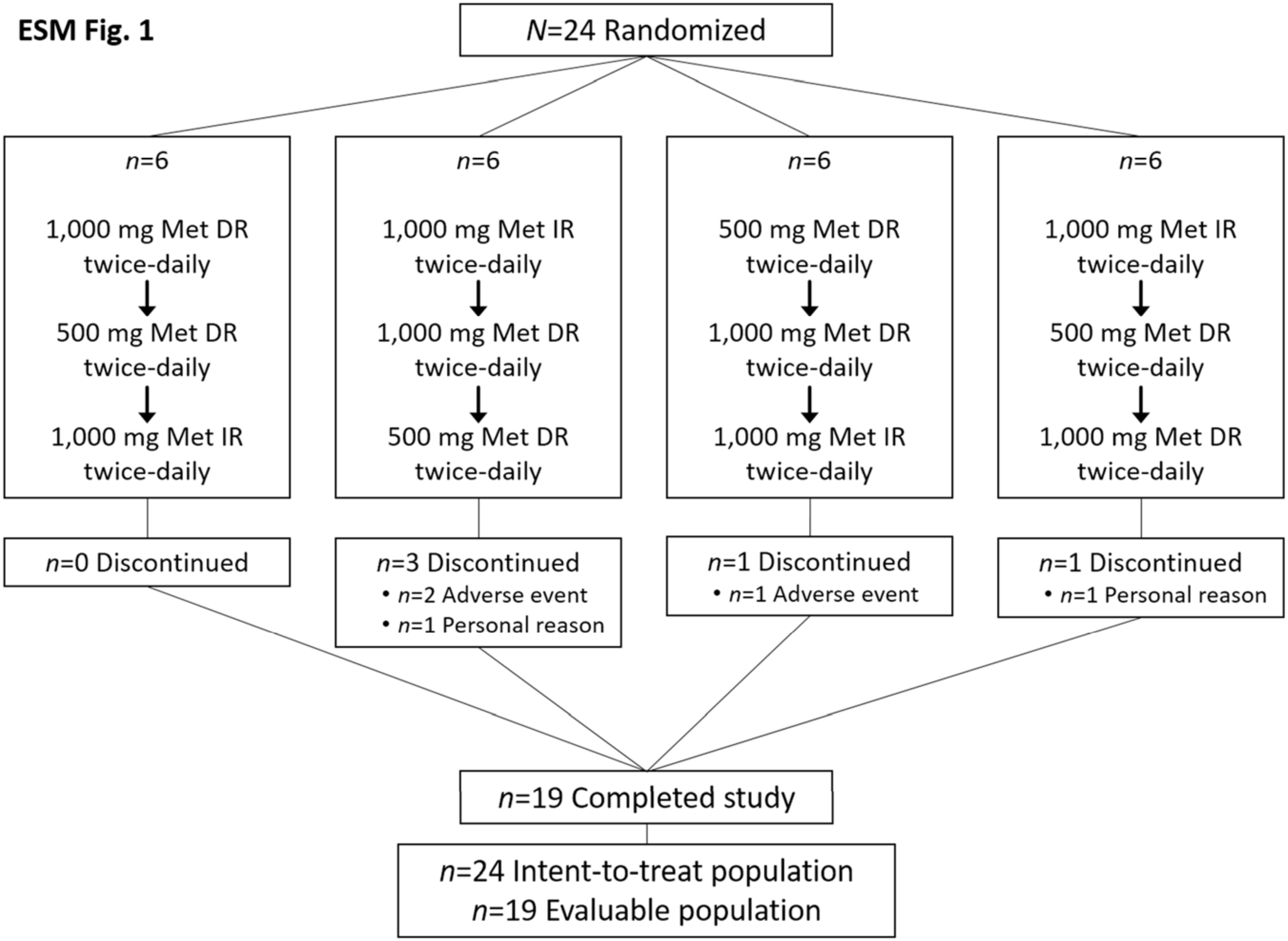
N=24 Randomized
n=6
1,000 mg Met DR twice-daily
500 mg Met DR twice-daily
1,000 mg Met IR twice-daily
n=6
1,000 mg Met IR twice-daily
1,000 mg Met DR twice-daily
500 mg Met DR twice-daily
n=6
500 mg Met DR twice-daily
1,000 mg Met DR twice-daily
1,000 mg Met IR twice-daily
n=6
1,000 mg Met IR twice-daily
500 mg Met DR twice-daily
1,000 mg Met DR twice-daily
n=0 Discontinued
n=3 Discontinued
• n=2 Adverse event
• n=1 Personal reason
n=1 Discontinued
• n=1 Adverse event
n=1 Discontinued
• n=1 Personal reason
n=19 Completed study
n=24 Intent-to-treat population
n=19 Evaluable population

ESM Fig. 2

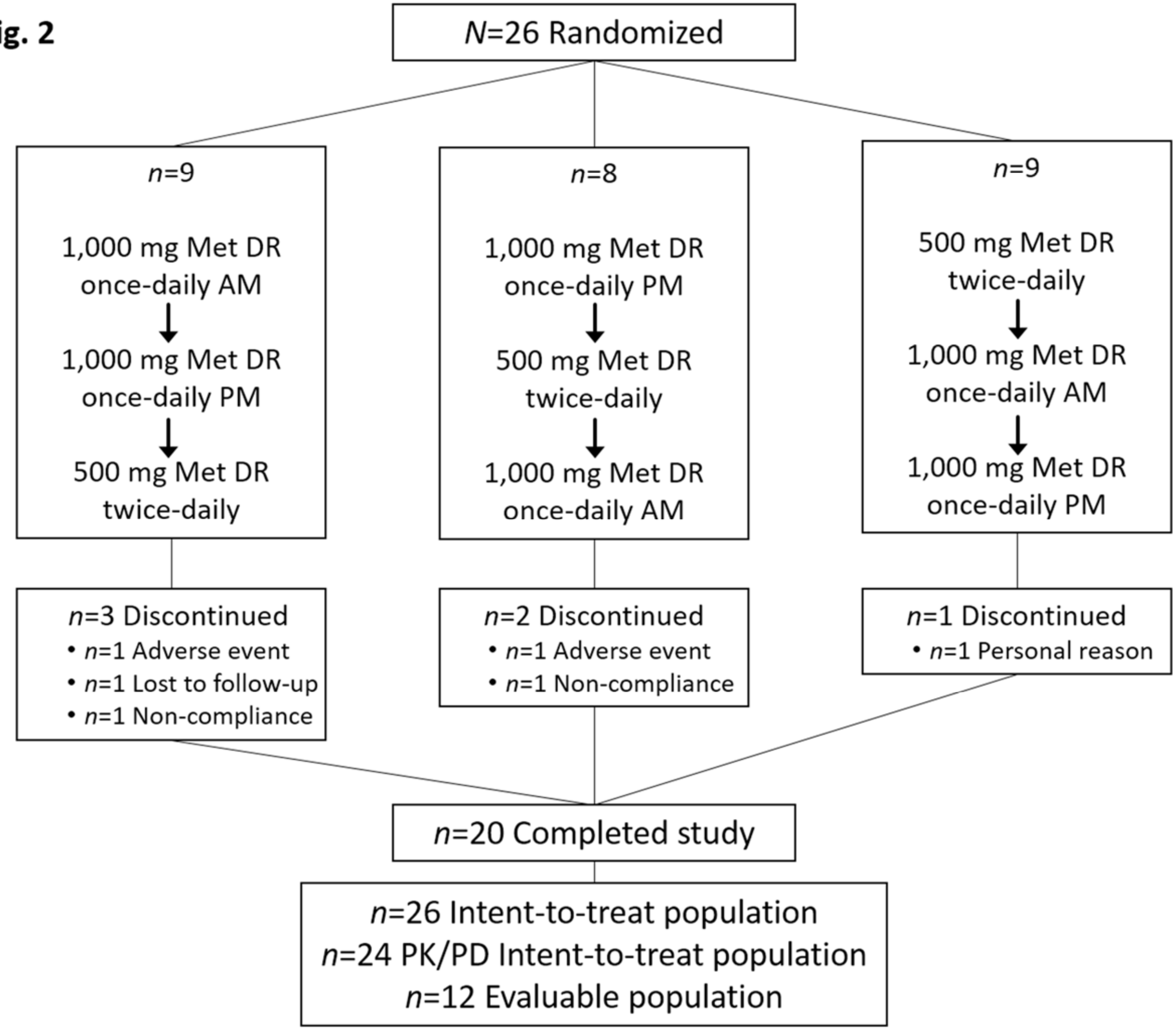

N=26 Randomized
n=9
1,000 mg Met DR once-daily AM
1,000 mg Met DR once-daily PM
500 mg Met DR twice-daily
n=8
1,000 mg Met DR once-daily PM
500 mg Met DR twice-daily
1,000 mg Met DR once-daily AM
n=9
500 mg Met DR twice-daily
1,000 mg Met DR once-daily AM
1,000 mg Met DR once-daily PM
n=3 Discontinued
• *n*=1 Adverse event
• *n*=1 Lost to follow-up
• *n*=1 Non-compliance
n=2 Discontinued
• *n*=1 Adverse event
• *n*=1 Non-compliance
n=1 Discontinued
• *n*=1 Personal reason
n=20 Completed study
n=26 Intent-to-treat population
n=24 PK/PD Intent-to-treat population
n=12 Evaluable population
